# Supplementary material for: Assessing the impact of the National Department of Health’s National Adherence Guidelines for Chronic Diseases in South Africa using routinely collected data: a cluster-randomised evaluation
Source: BMJ Open. 2018 Jan 21;8(1):e019680. doi: 10.1136/bmjopen-2017-019680 (PMC5781226; doi:10.1136/bmjopen-2017-019680)
Supplement: Author's manuscript [file bmjopen-2017-019680.draft_revisions.pdf]

BMJ Open

BMJ Open is committed to open peer review. As part of this commitment we make the peer review history of every article we publish publicly available.

When an article is published we post the peer reviewers' comments and the authors' responses online. We also post the versions of the paper that were used during peer review. These are the versions that the peer review comments apply to.

The versions of the paper that follow are the versions that were submitted during the peer review process. They are not the versions of record or the final published versions. They should not be cited or distributed as the published version of this manuscript.

BMJ Open is an open access journal and the full, final, typeset and author-corrected version of record of the manuscript is available on our site with no access controls, subscription charges or pay-per-view fees (http://bmjopen.bmj.com).

If you have any questions on BMJ Open's open peer review process please email editorial.bmjopen@bmj.com

BMJ Open

Evaluation of the National Department of Health's National Adherence Guidelines for Chronic Diseases in South Africa Using Routinely Collected Data: Protocol for a Randomised Evaluation

Journal:	*BMJ Open*
Manuscript ID	bmjopen-2017-019680
Article Type:	Protocol
Date Submitted by the Author:	20-Sep-2017

Complete List of Authors:	Fox, Matt; Boston University, Epidemiology and Global Health<br>Pascoe, Sophie; University of the Witwatersrand, Health Economics and Epidemiology Research Office, Department of Internal Medicine, School of Clinical Medicine, Faculty of Health Sciences<br>Huber, Amy; University of the Witwatersrand, Health Economics and Epidemiology Research Office, Department of Internal Medicine, School of Clinical Medicine, Faculty of Health Sciences<br>Murphy, Josh; University of the Witwatersrand, Health Economics and Epidemiology Research Office, Department of Internal Medicine, School of Clinical Medicine, Faculty of Health Sciences<br>Phokojoe, Mokgadi; National Department of Health, South Africa<br>Gorgens, Marelize; The World Bank Group<br>Rosen, Sydney; Boston University School of Public Health, Center for Global Health and Development<br>Wilson, David; The World Bank Group<br>Pillay, Yogan; National Department of Health, South Africa<br>Fraser-Hurt, Nicole; The World Bank Group

**Primary Subject Heading**:	HIV/AIDS
Secondary Subject Heading:	Global health, Health services research
Keywords:	HIV & AIDS < INFECTIOUS DISEASES, Adherence, Retention, Attrition, Viral Supression, Evaluation

SCHOLARONE™
Manuscripts

Evaluation of the National Department of Health's National Adherence Guidelines for Chronic Diseases in South Africa Using Routinely Collected Data: Protocol for a Randomised Evaluation

Matthew P Fox[1,2,3], Sophie Pascoe[2], Amy Huber[2], Joshua Murphy[2], Mokgadi Phokojoe[4], Marelize Gorgens[5], Sydney Rosen[1,2], David Wilson[5], Yogan Pillay[4], Nicole Fraser-Hurt[5]

1 Department of Global Health, Boston University School of Public Health, Boston, MA, USA

2 Health Economics and Epidemiology Research Office, Department of Internal Medicine, School of Clinical Medicine, Faculty of Health Sciences, University of the Witwatersrand

3 Department of Epidemiology, Boston University School of Public Health, Boston, MA, USA

4 National Department of Health, Pretoria, South Africa

5 The World Bank Group, Washington DC, USA

Word Count: 2879

Author for correspondence
Matthew Fox
mfox@bu.edu
Boston University School of Public Health
801 Massachusetts Ave
Boston MA 02118

For peer review only - http://bmjopen.bmj.com/site/about/guidelines.xhtml

Abstract

Introduction: In 2016, South Africa's National Department of Health (NDOH) launched the National Adherence Guidelines for Chronic Diseases for phased implementation throughout South Africa. Early implementation of a "minimum package" of eight interventions in the Adherence Guidelines for HIV patients is being undertaken at 12 primary health clinics and community health centers in four provinces. NDOH and its partners are evaluating the impact of five of the interventions in four provinces in South Africa.

Methods and analysis. The minimum package is being delivered at the 12 health facilities under NDOH guidance and through local health authorities. The five evaluation interventions are: 1) fast track initiation counseling for patients eligible for antiretroviral therapy (ART); 2) adherence clubs for stable ART patients; 3) decentralized medication delivery for stable ART patients; 4) enhanced adherence counseling for unstable ART patients; and 5) early tracing of patients who miss an appointment by ≥5 days. For evaluation, NDOH matched the 12 intervention clinics with 12 comparison clinics and randomly allocated one member of each pair to intervention or comparison (standard of care) status within pairs, allowing evaluation the interventions using a matched cluster-randomized design. The evaluation uses data routinely collected by the clinics, with no study interaction with subjects to prevent influencing the primary outcomes. Enrollment began on 20 June 2016 and was completed on 16 December 2016. A total of 3,456 patients were enrolled and will now be followed for 14 months to estimate effects on short- and long-term outcomes. Primary outcomes include viral suppression, retention and medication pick-ups, evaluated at two time points during follow up.

For peer review only - http://bmjopen.bmj.com/site/about/guidelines.xhtml

Ethics and dissemination. The study received approval from the University of Witwatersrand Human Research Ethics Committee and Boston University Institutional Review Board. Results will be presented to key stakeholders and at international conferences and published in peer-reviewed journals.

Strengths and limitations of this study

- Maintaining patient adherence to chronic disease medications, including HIV treatment, is a global challenge with a relatively weak implementation evidence base, making evaluation of adherence interventions essential.
- The evaluation assesses the impact of an adherence strategy that is planned for national implementation to improve adherence and retention and decongest clinics.
- The evaluation allows for rigorous evaluation and improvement of the interventions by utilizing a randomized rollout by the government with minimal burden on healthcare workers and patients.
- In order to prevent influencing retention outcomes, outcomes are collected through routine data monitoring systems, which contain missing data and classification errors.

For peer review only

INTRODUCTION

For antiretroviral therapy (ART) for HIV to be effective, patients must remain in care for long periods of time, initiate treatment as early as allowed under prevailing guidelines (now typically immediately after diagnosis in many countries), remain in care, consistently achieve high levels of adherence to their treatment regimen and, as a result, achieve and sustain an undetectable viral load. Treatment is lifelong and requires consistent, nearly complete daily adherence to be successful. In South Africa, where some 3.7 million individuals are now on ART[1], numerous studies and reviews[2–5] and the South African National Department of Health's (NDOH) own data[6] have indicated that retention in care and adherence to ART in South Africa are sub-optimal and pose a serious threat to the long-term success of the national HIV response.

To address this challenge, in 2014 the NDOH developed the "National Adherence Guidelines for Chronic Diseases (HIV, TB and NCDs)"[7,8]. The guidelines call for the provision of a minimum package of eight interventions to increase linkage to care, retention in care, and adherence to treatment. Although there is some published and unpublished evidence of the effectiveness of each of these interventions for HIV care[9–11], most have not been implemented jointly or at scale, nor have they been evaluated as delivered routinely by public sector facilities, without external technical assistance or resource support. Better information is needed to guide the NDOH's rollout of the minimum package at national scale and about the number of patients requiring each intervention.

Prior to national scale-up of the Adherence Guidelines, the NDOH selected 12 clinics (primary health care clinics and community health centres) for early implementation of the minimum package for HIV patients. This will generate information to refine the guidelines and gain experience in implementation.

For peer review only - http://bmjopen.bmj.com/site/about/guidelines.xhtml

This manuscript presents the protocol for a matched cluster-randomized evaluation to assess the impact of five of the interventions that are part of the National Adherence Guidelines on HIV retention and viral suppression outcomes at public sector clinics. The specific objectives of the evaluation are to evaluate the impact of:

1. Among HIV-infected patients newly eligible for antiretroviral therapy, *fast track treatment initiation counselling* on ART initiation and viral suppression.

2. Among HIV-infected patients who are stable on antiretroviral therapy, *adherence clubs* on ART adherence and viral suppression.

3. Among HIV-infected patients who are stable on antiretroviral therapy, *decentralized medication delivery* on ART adherence and viral suppression.

4. Among HIV-infected patients who have poor adherence (as indicated by an unsuppressed viral load) to antiretroviral therapy, *enhanced adherence counselling* on ART adherence and viral suppression.

5. Among HIV-infected patients in antiretroviral therapy programs who miss a scheduled appointment by 5 days, *early patient tracing* on retention in care.

METHODS AND ANALYSIS

For peer review only - http://bmjopen.bmj.com/site/about/guidelines.xhtml

Study Design

The study will estimate the effectiveness of each of five interventions included in the minimum package of adherence interventions using a matched cluster randomized design. It is taking place at 24 public sector clinics in four provinces in South Africa. The interventions were developed by South Africa's NDOH and are being implemented by the clinics using their own staff and resources; the study itself is providing no additional services. All non-pregnant adult patients seeking HIV-related services at the study sites and eligible to receive one of the interventions during the study enrollment period are eligible for inclusion in the study. The study has no direct interaction with study subjects and no study visits. Data are instead collected from routinely completed patient records, including clinic files, registers, and databases. Because the rollout was conducted by the NDOH and the interventions delivered by the sites, the study team did not have contact with individual patients. Instead the study was approved for analysis of data routinely collected by the study sites. As no patient contact occurred, we received a waiver of consent.

Interventions

The interventions in the minimum package are listed in Table 1. The following five interventions will be evaluated under this protocol. Each is described in detail in the National Adherence Guidelines, which are available at https://www.nacosa.org.za/wp-content/uploads/2016/11/Integrated-Adherence-Guidelines-NDOH.pdf [7] .

1. *Fast track initiation counseling (FTIC)* seeks to reduce attrition from chronic care by speeding up the process of treatment initiation for patients who are eligible for treatment and thereby increasing the proportion of treatment-eligible patients who start treatment promptly. For HIV,

For peer review only - http://bmjopen.bmj.com/site/about/guidelines.xhtml

the goal is to reduce the total number of visits that patients need to complete in order to start treatment and allow patients to initiate treatment over the course of two clinic visits within one week of confirming ART eligibility, with additional counseling provided in the first two routine visits after treatment initiation. The intervention includes a detailed curriculum for the counseling sessions, and providers work with patients to create an individualized adherence plan and ensure post-initiation adherence support[12].

2. *Adherence clubs (AC)* comprise adherent and stable patients on ART who meet at facilities or identified locations in the community, in groups of up to 30 patients every two to three months to receive group counseling, have a clinical assessment, and receive the required supply of pre-packed medications. Adherence clubs are facilitated by a nurse and lay staff at the health care facility with support from community health workers. The goal is to keep patients engaged in care and adherent to their medication by providing social support and facilitating medication delivery and treatment monitoring, while also reducing patient visit burden on the clinics[13]. The adherence guidelines SOP provides detailed instructions for establishing and running the clubs and for eligibility criteria and data collection.

3. *Decentralized medication delivery (DMD)* through the Central Chronic Medicine Dispensing and Distribution (CCMDD) and Chronic Dispensing Unit (CDU) programmes uses locations other than the clinic pharmacy to deliver medications to patients who are stable on treatment. Patients then only need to come to the clinic on a six monthly basis for a clinical exam. The goal is to reduce the burden on the patient in terms of the time and resources it takes them to collect their medication to improve treatment adherence and retention in care, while also decongesting the clinics.

For peer review only - http://bmjopen.bmj.com/site/about/guidelines.xhtml

4. *Enhanced adherence counseling (EAC)* seeks to identify patients who have poor treatment adherence as indicated by an elevated viral load and target these patients for enhanced adherence counseling to help them improve their adherence. Although HIV-infected patients with detectable viral loads may receive additional adherence counseling under the standard of care, this intervention will standardize and intensify that counseling. The intervention includes one or two structured education/counseling sessions in which effective strategies for achieving good adherence are discussed and goals set for viral re-suppression.

5. *Early tracing and retention in care of patients (TRIC)* who miss an appointment by 5 days or more seeks to identify patients who have not returned to the clinic for scheduled appointments and attempts to return them to care through contact by phones, text message and/or home visits. This intervention requires obtaining permission from patients to contact them and maintaining up to date contact information in patient records. The goal is to reduce clinic loss to follow up and improve patient outcomes by identifying those who have missed appointments and encouraging them to return to care.

Each of the interventions, while implemented as a package of services, is delivered to a unique population within each clinic: patients newly eligible for ART (FTIC), patients stable on ART (DMD or AC), patients with poor adherence (EAC), and patients lost from care (TRIC). Patients who are stable on ART can be provided with either decentralized medication delivery or adherence clubs, but not both. Because the patient populations differ, the study can estimate the effect of each intervention individually, in the context of implementation of the overall minimum package.

For peer review only - http://bmjopen.bmj.com/site/about/guidelines.xhtml

In order for the study to be as close as possible to routine care conditions the interventions for this study are being implemented by the study sites with no input or oversight from the study team. The interventions follow the National Adherence Guidelines and NDOH has organized trainings prior to the implementation of the interventions to support appropriate implementation of the guidelines.

Selection and Randomization of Study Sites

The evaluation is being conducted at 24 primary health care clinics (PHCs) in South Africa. All study sites follow the current guidelines for HIV care and treatment, dated December 2014[14] . Six clinics were chosen from one district each in Gauteng, KwaZulu-Natal, Limpopo, and North West Provinces. These provinces were chosen in consultation with NDOH to represent high HIV burden regions with high burden districts and high volume clinics. The study team developed a list of all sites in each participating province that met these criteria and selected three matched pairs of clinics per province. Pairs were matched on ART patient volume (1000-1999, 2000-4999, or ≥5000 current ART patients), setting (urban, informal settlement, or rural), location (pairs should be located relatively nearby one another), and HIV viral suppression rate (see Table 2). In each pair, one clinic was randomly assigned (using a computer generated randomization) to receive early implementation of the minimum package of interventions, while the other continued to provide standard of care. No blinding was used.

For peer review only

Inclusion and Exclusion Criteria

For each objective, we enrolled a specific cohort of patients as shown in Figure 1. All cohorts included patients aged 18 years or above and excluded patients who are not resident in the facility's catchment area, were recorded as having an intention to transfer care to a different facility within 12 months, or

For peer review only - http://bmjopen.bmj.com/site/about/guidelines.xhtml

were pregnant and eligible for PMTCT. Each cohort had specific inclusion criteria related to eligibility for the specific intervention. These criteria followed the December 2014 national guidelines for HIV care and ART[14] and July 2016 National Adherence Guidelines for Chronic Disease (HIV, TB and NCDs) [11]. In order to identify eligible patients to enroll, we first identified all patients eligible for each intervention based on information recorded on their electronic medical record. At intervention sites, lists were reviewed against clinic records, registers, and other documentation for each intervention, to identify eligible patients. At control sites, we reviewed lists against clinic records to confirm eligibility. If the patient file was found and eligibility for a cohort was confirmed then patients were enrolled sequentially until the required sample size was reached for that cohort. Due to delays in electronic data capturing data was not complete. To account for this at some sites, individuals receiving each intervention were identified directly from registers for that intervention. Clinic files were then reviewed to confirm eligibility and patients were enrolled up until the required sample size was achieved. For each patient enrolled, regardless of the method used to identify them, patient files were reviewed and information was extracted using an electronic case report form to confirm patients did meet all eligibility criteria for that cohort.

Duration of Follow Up

The study enrollment period began on 20 June 2016 and was completed on 16 December 2016. For individuals, observation began on the date of determination of eligibility for an intervention. Follow up of the cohorts is now ongoing and is anticipated to be completed in December of 2017. Passive follow up through medical record and database review will continue for a minimum of 14 months after the date of enrollment (two additional months beyond twelve months to allow one-year outcomes to occur and be recorded). This will allow all subjects sufficient follow up time to complete each of the primary

For peer review only

For peer review only - http://bmjopen.bmj.com/site/about/guidelines.xhtml

outcomes designated.

Data Sources

As noted, this study is relying on routinely collected data for study outcomes. Routine data sources will include TIER.Net, the National Health Laboratory Service (NHLS) database which contains all laboratory tests done in public-sector clinics, and data sets created by entering information from clinic registers, adherence plans, and patient clinic files into a database. Various degrees of strengthening of existing data collection procedures were needed at the facilities in order to ensure complete entries into existing clinic registers or patient files, complete and accurate entry of source data onto electronic files, and the use of a consistent clinic-level patient identifier to link patients between data sources (e.g. a register containing a row for each visit and a patient file containing documents pertaining to that patient will each contribute to the evaluation record for that patient).

Study Outcomes

Table 3 and Figure 1 list the primary and secondary outcomes we will measure for each of the objectives. Each primary outcome includes both a short-term (S) outcome and a longer-term (L) outcome for assessment of the immediate and longer-term effects of the intervention. Short-term outcomes are typically focused on retention-based outcomes within the first three to four months after the intervention, with the exception of FTIC in which we assess the impact on treatment initiation within the first month after eligibility. Long-term outcomes are focused mainly on retention and viral suppression at twelve months. Note that viral suppression in the current South African ART guidelines is defined as viral load below 400 copies/ml[3], and this is the threshold that South Africa's National Health

For peer review only - http://bmjopen.bmj.com/site/about/guidelines.xhtml

Laboratory Service reports.

For all outcomes, retention in care is defined as (1-% attrition), with attrition calculated as the sum of reported deaths, loss to follow up, and reported transfers to other facilities. Retention will thus be interpreted as “retained in care at facility,” since the outcomes of patients who transfer will not be known. Loss to follow up is defined as failure to attend the clinic within 90 days of a scheduled appointment, as stated in the Adherence Guidelines (Table 7 page 49).

Sample Size

Table 4 shows the sample size that is required to detect meaningful differences for Objectives 1-5. Sample sizes were determined using PASS software for cluster-randomized designs. Each sample size was determined to measure our short-term outcome for the objective. All calculations assume a site-clustered design with the clinic as the cluster and 24 clusters evenly split between intervention and comparison groups. We assumed power of 80% and an alpha of 0.05. Sample sizes accounted for the cluster randomized design by assuming a coefficient of variation of 0.1. Each sample size was calculated assuming a baseline proportion of patients achieving the outcome in the absence of the intervention as determined from the literature or experience. Sample sizes were calculated based on being able to detect an absolute increase on outcomes deemed to be clinically meaningful, ranging from 15% to 20%. The total sample size was calculated to be 3,456 including all of the five HIV cohorts.

For peer review only - http://bmjopen.bmj.com/site/about/guidelines.xhtml

Data Analysis

Our general analytic plan will be the same for each of the five interventions. We will begin with descriptive analyses of the characteristics of each of the cohorts stratified by intervention/non-intervention (comparison) status. We will also look for differences within the randomized matched pairs. Because the data will be collected as part of a clustered design, the data analysis will need to account for clustering. For each primary outcome described above, we will conduct a crude analysis comparing the proportion of subjects with the outcome in the intervention and comparison arms. Next, we will conduct an analysis for each outcome accounting only for clustering using generalized estimating equations (GEE) with an unstructured correlation matrix and clustering by treatment site. In all cases, the outcomes are dichotomous and therefore we will calculate relative risks or risk differences comparing the intervention to the comparison arms using a log (or identify) link function and a binomial distribution. Next, should any imbalances between treatment groups be detected, we will adjust for those covariates in our GEE model using covariate adjustment or difference in differences. Finally, we will look for differences in the effects of the strategies by important baseline characteristics (e.g. size of the treatment population, rural vs. urban, province, etc.) using stratified analyses.

Ethics and Dissemination

The study has received ethics approval from both the University of the Witwatersrand Human Research Ethics Committee (Medical) and the Boston University Institutional Review Board. In South Africa, we have also received national, provincial, and district-level approvals and the trial has been registered at clinicaltrials.gov (NCT02536768). Results of the study will be presented to key stakeholders as well as at international conferences and published in peer-reviewed journals.

For peer review only - http://bmjopen.bmj.com/site/about/guidelines.xhtml
For peer review only

The study team does not have any interaction with study subjects. Data for the study is drawn from existing records that are routinely collected at the study sites as part of routine patient care. We therefore believe that our study poses no physical risks to subjects. The only risk that we believe is posed by this study is that of loss of confidentiality. We are collecting data indicating individuals' HIV status and other sensitive health information. A high level of stigmatization continues to inhibit the disclosure of HIV status in the study population. A breach of confidentiality, for example through inadvertent loss of a storage device or paper files, would thus pose a risk to subjects.

We are protecting against the risk and repercussions of loss of confidentiality in two main ways. First, patient identifiers are stored separately from all other individual data in encrypted, password protected files. Analytic data sets will not contain any identifiers, and the linking files containing the identifiers will be destroyed once all linking has been accomplished. Second, all study data is stored in secure locations. Password-protected laptops and tablets used on site are kept in locked and secure locations when not in use. All data collected on tablets is immediately uploaded to a secure cloud server as soon as data collection for a patient is complete and is not kept on the tablets. Patient data extracted from electronic patient systems is extracted in a password protected double-encrypted format and uploaded to a secure server via a dedicated secure virtual private network. All study staff have been trained in Good Clinical Practice, Research Ethics, and study procedures to ensure that they understand both research confidentiality requirements and study confidentiality procedures. Study investigators monitor data collection on an ongoing basis.

For peer review only - http://bmjopen.bmj.com/site/about/guidelines.xhtml

We are not seeking informed consent for this study, which is a record review only and poses minimal risk to study subjects. The interventions have been provided by the study clinics as standard care under the early roll-out of NDOH's new Adherence Guidelines, not as part of the study itself.

Dissemination of Findings

The primary audience for this evaluation is the South African National Department of Health and its partners, which will use the results to improve, target, and budget for the national implementation of the Adherence Guidelines. Many of the findings, however, will likely be of broader interest in South Africa and other countries, where effective strategies for improving chronic disease medication adherence are eagerly sought. Results of the evaluation will be made as widely available as possible, through journals, websites, and conferences. Only aggregated, stratified data will be presented and it will not be possible to identify any individual patients from any of the data that is presented.

Limitations

While our study has benefits in terms of the cluster randomized approach and the fact that it was implemented at numerous sites around South Africa under routine conditions, it also has some important limitations. First, as we do not control the implementation of the interventions, we cannot ensure that they are followed according to guidelines. If they are implemented poorly, then effects will be biased towards no effect. Second as we do not control the data collection in the clinical files, we do have some missing and misclassified data. Third, because many of these interventions are improvements on previous approaches that are already part of guidelines (e.g. fast track initiation improves upon fast tracking of patients with low CD4 counts, etc.) we do not have pure control group. This would have the tendency of biasing towards no effect as well.

For peer review only - http://bmjopen.bmj.com/site/about/guidelines.xhtml

For peer review only

Conclusion

This study will be the first to evaluate the impact of a package of interventions that have been developed to improve adherence and retention in South Africa's National HIV Treatment Program. If these interventions are successful, they have the potential to improve outcomes on a national scale and potentially reduce HIV transmission. Thus, the results of this study should directly inform policy within South Africa and may be relevant to other countries in the region.

For peer review only

For peer review only - http://bmjopen.bmj.com/site/about/guidelines.xhtml

REFERENCES

1. SANAC and National Department of Health. South Africa's National Strategic Plan for HIV, TB and STIs 2017-2022. Pretoria, South Africa; 2017.
2. Rosen S, Fox MP. Retention on antiretroviral therapy in South Africa: evidence from a systematic review 2008-2013. Johannesburg; 2014. Report No.: 8.
3. Fox MP, Rosen S. Retention of Adult Patients on Antiretroviral Therapy in Low- -and Middle- -Income Countries: Systematic Review 2008. J Acquir Immune Defic Syndr. 2015;69: 98–108. doi:10.1097/QAI.0000000000000553
4. Fox MP, Shearer K, Maskew M, Meyer-Rath G, Clouse K, Sanne I. Attrition through Multiple Stages of Pre-Treatment and ART HIV Care in South Africa. PLoS One. 2014;9: e110252. doi:10.1371/journal.pone.0110252
5. Clouse K, Pettifor AE, Maskew M, Bassett J, Rie A Van, Behets F, et al. Patient Retention From HIV Diagnosis Through One Year on Antiretroviral Therapy at a Primary Health Care Clinic in Johannesburg , South Africa. J Acquir Immune Defic Syndr. 2013;62: 39–46.
6. DOH. Health Indicators Update- Antiretroviral Indictaors 2013. Pretoria; 2013.
7. National Department of Health. National adherence guidelines for chronic diseases (HIV, TB and NCDs), Version: 7 April 2015. Pretoria; 2015.
8. National Department of Health Republic of South Africa. Standard Operating Procedures for Minimum Package of Interventions to Suport Linkage to Care, Adherence and Retention in Care, Adherence Guidelines for HIV, TB and NCDs [Internet]. Pretoria, South Africa; 2016. Available: http://www.differentiatedcare.org/Portals/0/adam/Content/_YiT3_-qmECUkmkpQvZAIA/File/SOP A5 booklet 20-05-2016.pdf
9. World Bank, The World Bank. Evaluation of interventions to increase the proportion of people living with HIV who are diagnosed, initiated on, adhering to and retained in HIV treatment and care in South Africa. Formative Qualitative Research: Phase 1 Report. 2014.
10. Chaiyachati KH, Ogbuoji O, Price M, Suthar AB, Negussie EK, Bärnighausen T. Interventions to improve adherence to antiretroviral therapy: a rapid systematic review. AIDS. 2014;28 Suppl 2: S187-204. doi:10.1097/QAD.0000000000000252
11. Govindasamy D, Meghij J, Negussi EK, Baggaley RC, Ford N, Kranzer K. Interventions to improve or facilitate linkage to or retention in pre-ART (HIV) care and initiation of ART in low- and middle-income settings: a systematic review. J Int AIDS Soc. 2014;17: 19032.
12. Médecins Sans Frontières Khayelitsha. ART/TB/PMTCT initiation patient education and counselling model report and toolkit. Cape Town; 2015.
13. Medicins Sans Frontieres. ART adherence club report and toolkit. Cape Town; 2014.
14. National Department of Health. National consolidated guidelines for the prevention of mother-to-child transmission of HIV (PMTCT) and the management of HIV in children, adolescents and adults. Pretoria; 2014.
15. Rosen S, Maskew M, Fox MP, Nyoni C, Mongwenyana C, Malete G, et al. Initiating Antiretroviral Therapy for HIV at a Patient's First Clinic Visit: The RapIT Randomized Controlled Trial. Binagwaho A, editor. PLOS Med. 2016;13: e1002015. doi:10.1371/journal.pmed.1002015
16. Fox M, Maskew M, MacPhail A. Cohort Profile: The Themba Lethu Clinical Cohort, Johannesburg, South Africa. Int J Epidemiol. 2013;42: 430–439. doi:10.1093/ije/dys029
17. Fox M, Shearer K, Maskew M, Macleod W, Majuba P, Macphail P, et al. Treatment outcomes after 7 years of public-sector HIV treatment. AIDS. 2012;26: 1823–8. doi:10.1097/QAD.0b013e328357058a
18. Fox MP, Maskew M, Brennan AT, Evans D, Onoya D, Malete G, et al. Cohort profile: the Right to Care Clinical HIV Cohort, South Africa. BMJ Open. 2017;7: bmjopen-2016-015620. doi:10.1136/bmjopen-2016-015620

For peer review only - http://bmjopen.bmj.com/site/about/guidelines.xhtml

Authors' contributions: NF, MG, MP, SP, SR and MPF all contributed to developing the protocol. AH, JM, DW, MN and YP all contributed substantive changes to the protocol. MPF drafted the manuscript. All authors were involved in editing the final manuscript.

Funding statement: This work was supported by World Bank trust funds from several governments and Government of South Africa domestic health financing.

Competing interests statement: The authors declare that they have no competing interests, as the study funders played no role in the decision to publish this protocol.

For peer review only

Table 1. South Africa's National Adherence Guidelines minimum package of interventions

Approach	Intervention
Education and counselling	1. Fast track initiation counseling*<br>2. Enhanced adherence counseling for unstable patients*<br>3. Child disclosure counseling for children living with HIV
Repeat prescription collection strategies	4. Adherence clubs*<br>5. Spaced and fast lane appointment systems<br>6. Decentralised medication delivery*
Patient tracing	7. Early tracing of all missed appointments*
Integrated HIV, TB, NCD care	8. Integrated consultation and counselling

*Indicates interventions included in this evaluation

For peer review only

For peer review only - http://bmjopen.bmj.com/site/about/guidelines.xhtml

Table 2: Location of early learning sites, allocation status and value of matching variables used to determine matched pairs*

District & Province	Pair	Site number	Sub-district [1]	Study allocation	Total remaining on ART (Nov 2014)	% of viral loads where VL<400 copies/ml [2]
Ekurhuleni, Gauteng	1	1	S2	Intervention	1094	71%
		2	S2	Control	1098	66%
	2	3	S2	Intervention	2676	86%
		4	S2	Control	2749	76%
	3	5	S2	Intervention	1929	84%
		6	S2	Control	1072	80%
Mopani, Limpopo	4	7	Greater Tzaneen	Intervention	1720	71%
		8	Greater Tzaneen	Control	1022	64%
	5	9	Greater Giyani	Intervention	1702	73%
		10	Greater Giyani	Control	1445	76%
	6	11	Greater Tzaneen	Intervention	1370	77%
		12	Greater Tzaneen	Control	1027	76%
Bojanala Platinum, North West	7	13	Madibeng	Intervention	4147	83%
		14	Madibeng	Control	4182	82%
	8	15	Madibeng	Intervention	1152	83%
		16	Madibeng	Control	1224	81%
	9	17	Rustenburg	Intervention	3951	80%
		18	Rustenburg	Control	3328	78%
King Cetshwayo (previously uThungulu), KwaZulu Natal	10	19	uMlalazi	Intervention	1900	72%
		20	uMlalazi	Control	1053	69%
	11	21	uMhlathuze	Intervention	5037	83%
		22	uMhlathuze	Control	7305	82%
	12	23	Ntambanana	Intervention	1111	81%
		24	Ntambanana	Control	1184	88%

[1] Used as proxy for setting and location

[2] NHLS data April 2014 to March 2015

* Data source: MacLeod, W., Bor, J., Crawford, K., & Carmona, S. (2015). Analysis of Big Data for better targeting of ART Adherence Strategies: Spatial clustering analysis of viral load suppression by South African province, district, sub-district and facility (April 2014-March 2015). Department of Health, Pretoria, South Africa.

For peer review only - http://bmjopen.bmj.com/site/about/guidelines.xhtml

Table 3. Short-term (S) and long-term (L) evaluation outcomes for the Adherence Guideline impact evaluation in South Africa

Objective	Primary Outcome	Secondary outcomes
Fast track ART initiation counseling (Objective/Cohort 1)	Proportion of patients who initiate ART within 30 days of becoming ART eligible (S) and the proportion of patients who are alive, in care, and virally suppressed (< 400 copies/ml^3) within nine months of ART eligibility (L).	Proportion of patients who initiate ART within one week of becoming ART eligible<br><br>Demographic and clinical characteristics of patients who do and do not achieve primary outcomes (age, sex, baseline CD4 counts, TB diagnosis, other characteristics as allowed by data).
Adherence clubs (Objective/Cohort 2)	Proportion of patients eligible for participation in an adherence club who receive all medications within the first four months after club eligibility (S) and the proportion virally suppressed (< 400 copies/ml^3) at twelve months after club eligibility (L).	Proportion of patients consistently participating in club<br><br>Demographic and clinical characteristics of patients who do and do not achieve primary outcomes.
Decentralized medication delivery (Objective/Cohort 3)	Proportion of patients eligible for decentralized medication delivery who receive all medications within the first three (S) months after delivery eligibility and viral suppression (< 400 copies/ml^3) twelve months after delivery eligibility (L).	Proportion of patients consistently receiving medications<br><br>Demographic and clinical characteristics of patients who do and do not achieve primary outcomes.
Enhanced adherence counseling (Objective/Cohort 4)	Proportion of patients with an elevated viral load who are alive, retained in care and resuppress their viral load (< 400copies/ml^3) within three (S) and twelve months (L) of eligibility for enhanced adherence counseling.	Demographic and clinical characteristics of patients who do and do not achieve primary outcomes.
Early tracing of patients lost to follow up (Objective/Cohort 5)	Proportion of patients eligible for early patient tracing who return to care within three (S) and twelve (L) months of eligibility.	Proportion of patients reached by tracers<br><br>Number of tracing attempts required; proportion of patients retained in care for at least one additional routine visit after tracing<br><br>Demographic and clinical characteristics of patients who do and do not achieve primary outcomes.

For peer review only

For peer review only - http://bmjopen.bmj.com/site/about/guidelines.xhtml

Table 4. Sample sizes for each objective of the Adherence Guideline impact evaluation study in South Africa

Objective	Sample Size	Rationale
Objective 1—Fast Track ART Initiation Counseling	720 patients	The RapIT study of rapid ART initiation[15], conducted at a well-managed PHC in Gauteng Province, found that about 60% of ART-eligible patients initiated under standard care within 30 days. Conservatively assuming 60% initiation without the intervention and 75% with the intervention, 30 subjects in each of the 24 clusters for 720 total subjects will be required to detect a difference of 15%. We have increased this by 20% to account for ineligible patients.
Objective 2—Adherence Clubs	576 patients	Data from Themba Lethu Clinic[16–18] show that about 80% of patients made all of their medication pickups over a three month period. It is anticipated that 24 subjects per clinic for a total of 576 patients will be needed to detect a difference of 15%. We have increased this by 20% to account for ineligible patients.
Objective 3—Decentralized Medication Delivery	576 patients	Data from Themba Lethu Clinic[16–18] show about 80% of patients made all of their medication pickups over a three month period. It is anticipated that 24 subjects per clinic for a total of 576 patients will be needed to detect a difference of 15%. We have increased this by 20% to account for ineligible patients.
Objective 4—Enhanced Adherence Counseling	1008 patients	Data from KwaZulu-Natal Province indicate that 52% of patients with a detectable viral load re-suppress after one session. It is anticipated that 42 subjects per clinic for a total of 1008 patients will be needed to detect a difference of 15%. We have increased this by 20% to account for ineligible patients.
Objective 5—Early tracing of patients lost to follow up	576 patients	Data from various Right to Care clinics[18] suggest that the proportion of patients who are lost from care who return with no or little intervention is low, between 20-35%. It is anticipated that 24 subjects per clinic for a total of 576 patients will be needed to detect a difference of 15% assuming a baseline of 30% loss to follow up without intervention. We have increased this by 20% to account for ineligible patients.

For peer review only

For peer review only - http://bmjopen.bmj.com/site/about/guidelines.xhtml

Figure 1 – Eligible population for each evaluation cohort and short and long-term endpoints for the impact evaluation

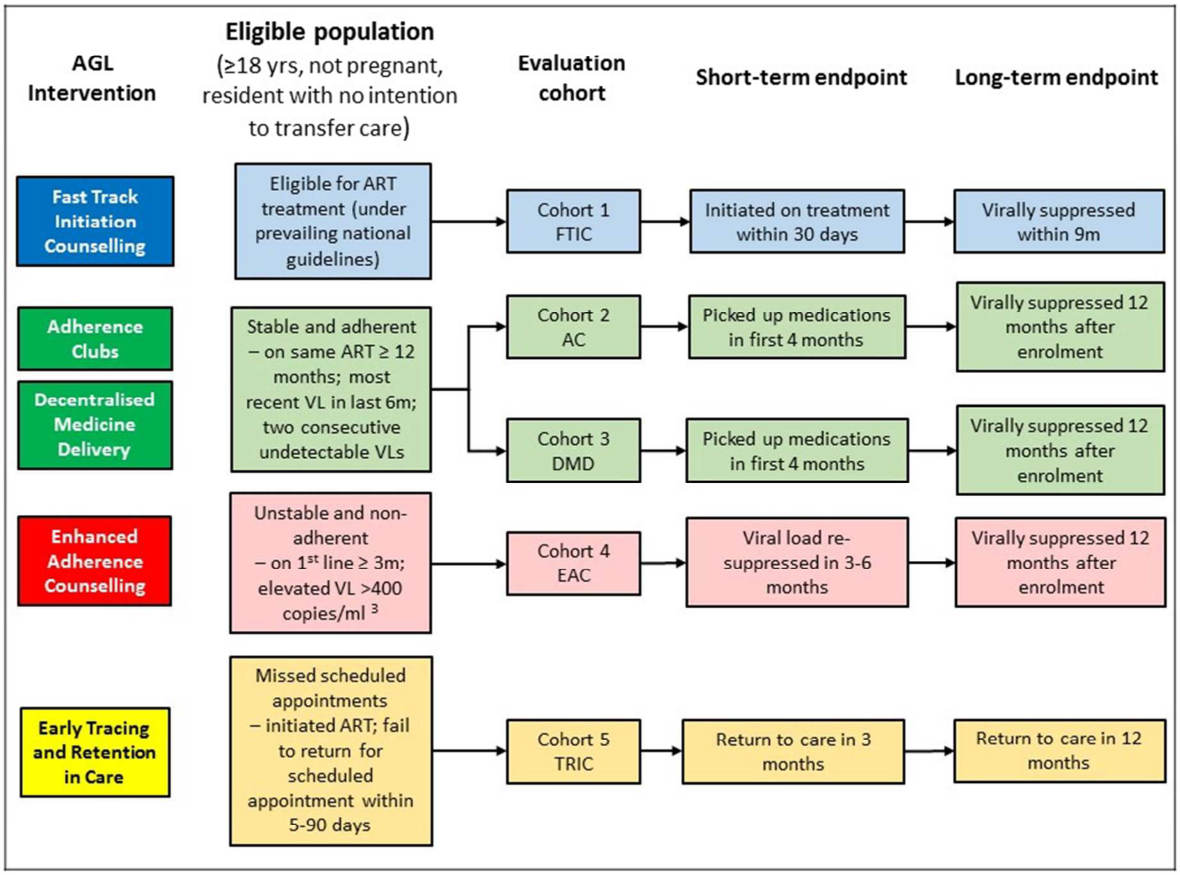

For peer review only

For peer review only - http://bmjopen.bmj.com/site/about/guidelines.xhtml

SPIRIT

STANDARD PROTOCOL ITEMS: RECOMMENDATIONS FOR INTERVENTIONAL TRIALS

SPIRIT 2013 Checklist: Recommended items to address in a clinical trial protocol and related documents*

Section/item	Item No	Description	Addressed on page number
**Administrative information**			
Title	1	Descriptive title identifying the study design, population, interventions, and, if applicable, trial acronym	_____1_______
Trial registration	2a	Trial identifier and registry name. If not yet registered, name of intended registry	_____13______
	2b	All items from the World Health Organization Trial Registration Data Set	_____Multiple__
Protocol version	3	Date and version identifier	_Protocol available
Funding	4	Sources and types of financial, material, and other support	Funding statement 18
Roles and responsibilities	5a	Names, affiliations, and roles of protocol contributors	1
	5b	Name and contact information for the trial sponsor	1
	5c	Role of study sponsor and funders, if any, in study design; collection, management, analysis, and interpretation of data; writing of the report; and the decision to submit the report for publication, including whether they will have ultimate authority over any of these activities	_18__________
	5d	Composition, roles, and responsibilities of the coordinating centre, steering committee, endpoint adjudication committee, data management team, and other individuals or groups overseeing the trial, if applicable (see Item 21a for data monitoring committee)	__n/a_________

For peer review only - http://bmjopen.bmj.com/site/about/guidelines.xhtml

For peer review only

Introduction

Background and rationale	6a	Description of research question and justification for undertaking the trial, including summary of relevant studies (published and unpublished) examining benefits and harms for each intervention	____4-5_____
	6b	Explanation for choice of comparators	____6-8_____
Objectives	7	Specific objectives or hypotheses	____4-5______
Trial design	8	Description of trial design including type of trial (eg, parallel group, crossover, factorial, single group), allocation ratio, and framework (eg, superiority, equivalence, noninferiority, exploratory)	_____6_______

Methods: Participants, interventions, and outcomes

Study setting	9	Description of study settings (eg, community clinic, academic hospital) and list of countries where data will be collected. Reference to where list of study sites can be obtained	____4 and 6__
Eligibility criteria	10	Inclusion and exclusion criteria for participants. If applicable, eligibility criteria for study centres and individuals who will perform the interventions (eg, surgeons, psychotherapists)	______9-10____
Interventions	11a	Interventions for each group with sufficient detail to allow replication, including how and when they will be administered	________6-8___
	11b	Criteria for discontinuing or modifying allocated interventions for a given trial participant (eg, drug dose change in response to harms, participant request, or improving/worsening disease)	____n/a______
	11c	Strategies to improve adherence to intervention protocols, and any procedures for monitoring adherence (eg, drug tablet return, laboratory tests)	____ n/a ______
	11d	Relevant concomitant care and interventions that are permitted or prohibited during the trial	_____ n/a ____
Outcomes	12	Primary, secondary, and other outcomes, including the specific measurement variable (eg, systolic blood pressure), analysis metric (eg, change from baseline, final value, time to event), method of aggregation (eg, median, proportion), and time point for each outcome. Explanation of the clinical relevance of chosen efficacy and harm outcomes is strongly recommended	_____11-12____
Participant timeline	13	Time schedule of enrolment, interventions (including any run-ins and washouts), assessments, and visits for participants. A schematic diagram is highly recommended (see Figure)	____9-10______

For peer review only - http://bmjopen.bmj.com/site/about/guidelines.xhtml

Sample size	14	Estimated number of participants needed to achieve study objectives and how it was determined, including clinical and statistical assumptions supporting any sample size calculations	___12-13_____
Recruitment	15	Strategies for achieving adequate participant enrolment to reach target sample size	____13______
**Methods: Assignment of interventions (for controlled trials)**			
Allocation:			
Sequence generation	16a	Method of generating the allocation sequence (eg, computer-generated random numbers), and list of any factors for stratification. To reduce predictability of a random sequence, details of any planned restriction (eg, blocking) should be provided in a separate document that is unavailable to those who enrol participants or assign interventions	_____9______
Allocation concealment mechanism	16b	Mechanism of implementing the allocation sequence (eg, central telephone; sequentially numbered, opaque, sealed envelopes), describing any steps to conceal the sequence until interventions are assigned	_____n/a____
Implementation	16c	Who will generate the allocation sequence, who will enrol participants, and who will assign participants to interventions	_____9______
Blinding (masking)	17a	Who will be blinded after assignment to interventions (eg, trial participants, care providers, outcome assessors, data analysts), and how	_____9______
	17b	If blinded, circumstances under which unblinding is permissible, and procedure for revealing a participant's allocated intervention during the trial	_______n/a____
**Methods: Data collection, management, and analysis**			
Data collection methods	18a	Plans for assessment and collection of outcome, baseline, and other trial data, including any related processes to promote data quality (eg, duplicate measurements, training of assessors) and a description of study instruments (eg, questionnaires, laboratory tests) along with their reliability and validity, if known. Reference to where data collection forms can be found, if not in the protocol	_____13______
	18b	Plans to promote participant retention and complete follow-up, including list of any outcome data to be collected for participants who discontinue or deviate from intervention protocols	_____n/a_____

For peer review only - http://bmjopen.bmj.com/site/about/guidelines.xhtml

Data management	19	Plans for data entry, coding, security, and storage, including any related processes to promote data quality (eg, double data entry; range checks for data values). Reference to where details of data management procedures can be found, if not in the protocol	_____11,13____
Statistical methods	20a	Statistical methods for analysing primary and secondary outcomes. Reference to where other details of the statistical analysis plan can be found, if not in the protocol	____13_____
	20b	Methods for any additional analyses (eg, subgroup and adjusted analyses)	____13______
	20c	Definition of analysis population relating to protocol non-adherence (eg, as randomised analysis), and any statistical methods to handle missing data (eg, multiple imputation)	_____n/a_____
**Methods: Monitoring**			
Data monitoring	21a	Composition of data monitoring committee (DMC); summary of its role and reporting structure; statement of whether it is independent from the sponsor and competing interests; and reference to where further details about its charter can be found, if not in the protocol. Alternatively, an explanation of why a DMC is not needed	_____ n/a _____
	21b	Description of any interim analyses and stopping guidelines, including who will have access to these interim results and make the final decision to terminate the trial	_____ n/a _____
Harms	22	Plans for collecting, assessing, reporting, and managing solicited and spontaneously reported adverse events and other unintended effects of trial interventions or trial conduct	_______ n/a _____
Auditing	23	Frequency and procedures for auditing trial conduct, if any, and whether the process will be independent from investigators and the sponsor	_______ n/a _____
**Ethics and dissemination**			
Research ethics approval	24	Plans for seeking research ethics committee/institutional review board (REC/IRB) approval	_____13_____
Protocol amendments	25	Plans for communicating important protocol modifications (eg, changes to eligibility criteria, outcomes, analyses) to relevant parties (eg, investigators, REC/IRBs, trial participants, trial registries, journals, regulators)	_____13-14__

For peer review only - http://bmjopen.bmj.com/site/about/guidelines.xhtml

Consent or assent	26a	Who will obtain informed consent or assent from potential trial participants or authorised surrogates, and how (see Item 32)	___n/a______
	26b	Additional consent provisions for collection and use of participant data and biological specimens in ancillary studies, if applicable	_____ n/a_____
Confidentiality	27	How personal information about potential and enrolled participants will be collected, shared, and maintained in order to protect confidentiality before, during, and after the trial	_____14______
Declaration of interests	28	Financial and other competing interests for principal investigators for the overall trial and each study site	_____18______
Access to data	29	Statement of who will have access to the final trial dataset, and disclosure of contractual agreements that limit such access for investigators	___data agreement___
Ancillary and post-trial care	30	Provisions, if any, for ancillary and post-trial care, and for compensation to those who suffer harm from trial participation	____n/a_______
Dissemination policy	31a	Plans for investigators and sponsor to communicate trial results to participants, healthcare professionals, the public, and other relevant groups (eg, via publication, reporting in results databases, or other data sharing arrangements), including any publication restrictions	____15_______
	31b	Authorship eligibility guidelines and any intended use of professional writers	____n/a_______
	31c	Plans, if any, for granting public access to the full protocol, participant-level dataset, and statistical code	Declarations
**Appendices**			
Informed consent materials	32	Model consent form and other related documentation given to participants and authorised surrogates	n/a
Biological specimens	33	Plans for collection, laboratory evaluation, and storage of biological specimens for genetic or molecular analysis in the current trial and for future use in ancillary studies, if applicable	n/a

*It is strongly recommended that this checklist be read in conjunction with the SPIRIT 2013 Explanation & Elaboration for important clarification on the items. Amendments to the protocol should be tracked and dated. The SPIRIT checklist is copyrighted by the SPIRIT Group under the Creative Commons "Attribution-NonCommercial-NoDerivs 3.0 Unported" license.

For peer review only - http://bmjopen.bmj.com/site/about/guidelines.xhtml

BMJ Open

Assessing the Impact of the National Department of Health's National Adherence Guidelines for Chronic Diseases in South Africa Using Routinely Collected Data: A Cluster-Randomised Evaluation

Journal:	*BMJ Open*
Manuscript ID	bmjopen-2017-019680.R1
Article Type:	Protocol
Date Submitted by the Author:	01-Nov-2017

Complete List of Authors:	Fox, Matt; Boston University, Epidemiology and Global Health<br>Pascoe, Sophie; University of the Witwatersrand, Health Economics and Epidemiology Research Office, Department of Internal Medicine, School of Clinical Medicine, Faculty of Health Sciences<br>Huber, Amy; University of the Witwatersrand, Health Economics and Epidemiology Research Office, Department of Internal Medicine, School of Clinical Medicine, Faculty of Health Sciences<br>Murphy, Josh; University of the Witwatersrand, Health Economics and Epidemiology Research Office, Department of Internal Medicine, School of Clinical Medicine, Faculty of Health Sciences<br>Phokojoe, Mokgadi; National Department of Health, South Africa<br>Gorgens, Marelize; The World Bank Group<br>Rosen, Sydney; Boston University School of Public Health, Center for Global Health and Development<br>Wilson, David; The World Bank Group<br>Pillay, Yogan; National Department of Health, South Africa<br>Fraser-Hurt, Nicole; The World Bank Group

**Primary Subject Heading**:	HIV/AIDS
Secondary Subject Heading:	Global health, Health services research
Keywords:	HIV & AIDS < INFECTIOUS DISEASES, Adherence, Retention, Attrition, Viral Supression, Evaluation

SCHOLARONE™ Manuscripts

Assessing the Impact of the National Department of Health's National Adherence Guidelines for Chronic Diseases in South Africa Using Routinely Collected Data: A Cluster-Randomised Evaluation

Matthew P Fox[1,2,3], Sophie Pascoe[2], Amy Huber[2], Joshua Murphy[2], Mokgadi Phokojoe[4], Marelize Gorgens[5], Sydney Rosen[1,2], David Wilson[5], Yogan Pillay[4], Nicole Fraser-Hurt[5]

1 Department of Global Health, Boston University School of Public Health, Boston, MA, USA

2 Health Economics and Epidemiology Research Office, Department of Internal Medicine, School of Clinical Medicine, Faculty of Health Sciences, University of the Witwatersrand

3 Department of Epidemiology, Boston University School of Public Health, Boston, MA, USA

4 National Department of Health, Pretoria, South Africa

5 The World Bank Group, Washington DC, USA

Word Count: 2879

Author for correspondence
Matthew Fox
mfox@bu.edu
Boston University School of Public Health
801 Massachusetts Ave
Boston MA 02118

For peer review only

Abstract

Introduction: In 2016, South Africa's National Department of Health (NDOH) launched the National Adherence Guidelines for Chronic Diseases for phased implementation throughout South Africa. Early implementation of a "minimum package" of eight interventions in the Adherence Guidelines for HIV patients is being undertaken at 12 primary health clinics and community health centers in four provinces. NDOH and its partners are evaluating the impact of five of the interventions in four provinces in South Africa.

Methods and analysis. The minimum package is being delivered at the 12 health facilities under NDOH guidance and through local health authorities. The five evaluation interventions are: 1) fast track initiation counseling for patients eligible for antiretroviral therapy (ART); 2) adherence clubs for stable ART patients; 3) decentralized medication delivery for stable ART patients; 4) enhanced adherence counseling for unstable ART patients; and 5) early tracing of patients who miss an appointment by ≥5 days. For evaluation, NDOH matched the 12 intervention clinics with 12 comparison clinics and randomly allocated one member of each pair to intervention or comparison (standard of care) status within pairs, allowing evaluation the interventions using a matched cluster-randomized design. The evaluation uses data routinely collected by the clinics, with no study interaction with subjects to prevent influencing the primary outcomes. Enrollment began on 20 June 2016 and was completed on 16 December 2016. A total of 3,456 patients were enrolled and will now be followed for 14 months to estimate effects on short-term and final outcomes. Primary outcomes include viral suppression, retention and medication pick-ups, evaluated at two time points during follow up.

For peer review only - http://bmjopen.bmj.com/site/about/guidelines.xhtml

Ethics and dissemination. The study received approval from the University of Witwatersrand Human Research Ethics Committee and Boston University Institutional Review Board. Results will be presented to key stakeholders and at international conferences and published in peer-reviewed journals.

Strengths and limitations of this study

- The evaluation assesses the impact of an adherence strategy that is planned for national implementation to improve adherence and retention and decongest clinics.
- The evaluation allows for rigorous evaluation and improvement of the interventions by utilizing a randomized rollout by the government with minimal burden on healthcare workers and patients.
- However, as we do not control the implementation of the interventions, we cannot ensure that they are followed according to guidelines. Further if patients self-select into the interventions, this could also create some selection bias.
- In addition, as we do not control the data collection in the clinical files, we do have some missing and misclassified data and lack of blinding could lead to some misclassification.
- Finally, as many of these interventions are improvements on previous approaches that are already part of guidelines we do not have pure control group.

For peer review only

INTRODUCTION

For antiretroviral therapy (ART) for HIV to be effective, patients must remain in care for long periods of time, initiate treatment as early as allowed under prevailing guidelines (now typically immediately after diagnosis in many countries), remain in care, consistently achieve high levels of adherence to their treatment regimen and, as a result, achieve and sustain an undetectable viral load. Treatment is lifelong and requires consistent, nearly complete daily adherence to be successful. In South Africa, where some 3.7 million individuals are now on ART[1], numerous studies and reviews[2–5] and the South African National Department of Health's (NDOH) own data[6] have indicated that retention in care and adherence to ART in South Africa are sub-optimal and pose a serious threat to the long-term success of the national HIV response.

To address this challenge, in 2014 the NDOH developed the "National Adherence Guidelines for Chronic Diseases (HIV, TB and NCDs)"[7,8]. The guidelines call for the provision of a minimum package of eight interventions to increase linkage to care, retention in care, and adherence to treatment. Although there is some published and unpublished evidence of the effectiveness of each of these interventions for HIV care[9–11], most have not been implemented jointly or at scale, nor have they been evaluated as delivered routinely by public sector facilities, without external technical assistance or resource support. Better information is needed to guide the NDOH's rollout of the minimum package at national scale and about the number of patients requiring each intervention.

Prior to national scale-up of the Adherence Guidelines, the NDOH selected 12 clinics (primary health care clinics and community health centres) for early implementation of the minimum package for HIV patients. This will generate information to refine the guidelines and gain experience in implementation.

For peer review only - http://bmjopen.bmj.com/site/about/guidelines.xhtml

This manuscript presents the protocol for a matched cluster-randomized evaluation to assess the impact of five of the interventions that are part of the National Adherence Guidelines on HIV retention and viral suppression outcomes at public sector clinics. The specific objectives of the evaluation are to evaluate the impact of:

1. Among HIV-infected patients newly eligible for antiretroviral therapy, *fast track treatment initiation counselling* on ART initiation and viral suppression.

2. Among HIV-infected patients who are stable on antiretroviral therapy, *adherence clubs* on ART adherence and viral suppression.

3. Among HIV-infected patients who are stable on antiretroviral therapy, *decentralized medication delivery* on ART adherence and viral suppression.

4. Among HIV-infected patients who have poor adherence (as indicated by an unsuppressed viral load) to antiretroviral therapy, *enhanced adherence counselling* on ART adherence and viral suppression.

5. Among HIV-infected patients in antiretroviral therapy programs who miss a scheduled appointment by 5 days, *early patient tracing* on retention in care.

METHODS AND ANALYSIS

For peer review only - http://bmjopen.bmj.com/site/about/guidelines.xhtml
For peer review only

Study Design

The study will estimate the effectiveness of each of five interventions included in the minimum package of adherence interventions using a matched cluster randomized design. It is taking place at 24 public sector clinics in four provinces in South Africa. The interventions were developed by South Africa's NDOH and are being implemented by the clinics using their own staff and resources; the study itself is providing no additional services. All non-pregnant adult patients seeking HIV-related services at the study sites and eligible to receive one of the interventions during the study enrollment period are eligible for inclusion in the study. The study has no direct interaction with study subjects and no study visits. Data are instead collected from routinely completed patient records, including clinic files, registers, and databases. Because the rollout was conducted by the NDOH and the interventions delivered by the sites, the study team did not have contact with individual patients. Instead the study was approved for analysis of data routinely collected by the study sites. As no patient contact occurred, we received a waiver of consent.

Interventions

The interventions in the minimum package are listed in Table 1. The following five interventions will be evaluated under this protocol. Each is described in detail in the National Adherence Guidelines, which are available at https://www.nacosa.org.za/wp-content/uploads/2016/11/Integrated-Adherence-Guidelines-NDOH.pdf [7] .

1. *Fast track initiation counseling (FTIC)* seeks to reduce attrition from chronic care by speeding up the process of treatment initiation for patients who are eligible for treatment and thereby increasing the proportion of treatment-eligible patients who start treatment promptly. For HIV,

For peer review only - http://bmjopen.bmj.com/site/about/guidelines.xhtml

the goal is to reduce the total number of visits that patients need to complete in order to start treatment and allow patients to initiate treatment over the course of two clinic visits within one week of confirming ART eligibility, with additional counseling provided in the first two routine visits after treatment initiation. The intervention includes a detailed curriculum for the counseling sessions, and providers work with patients to create an individualized adherence plan and ensure post-initiation adherence support[12].

2. *Adherence clubs (AC)* comprise adherent and stable patients on ART who meet at facilities or identified locations in the community, in groups of up to 30 patients every two to three months to receive group counseling, have a clinical assessment, and receive the required supply of pre-packed medications. Adherence clubs are facilitated by a nurse and lay staff at the health care facility with support from community health workers. The goal is to keep patients engaged in care and adherent to their medication by providing social support and facilitating medication delivery and treatment monitoring, while also reducing patient visit burden on the clinics[13]. The adherence guidelines SOP provides detailed instructions for establishing and running the clubs and for eligibility criteria and data collection.

3. *Decentralized medication delivery (DMD)* through the Central Chronic Medicine Dispensing and Distribution (CCMDD) and Chronic Dispensing Unit (CDU) programmes uses locations other than the clinic pharmacy to deliver medications to patients who are stable on treatment. Patients then only need to come to the clinic on a six monthly basis for a clinical exam. The goal is to reduce the burden on the patient in terms of the time and resources it takes them to collect their medication to improve treatment adherence and retention in care, while also decongesting the clinics.

For peer review only

4. *Enhanced adherence counseling (EAC)* seeks to identify patients who have poor treatment adherence as indicated by an elevated viral load and target these patients for enhanced adherence counseling to help them improve their adherence. Although HIV-infected patients with detectable viral loads may receive additional adherence counseling under the standard of care, this intervention will standardize and intensify that counseling. The intervention includes one or two structured education/counseling sessions in which effective strategies for achieving good adherence are discussed and goals set for viral re-suppression.

5. *Early tracing and retention in care of patients (TRIC)* who miss an appointment by 5 days or more seeks to identify patients who have not returned to the clinic for scheduled appointments and attempts to return them to care through contact by phones, text message and/or home visits. This intervention requires obtaining permission from patients to contact them and maintaining up to date contact information in patient records. The goal is to reduce clinic loss to follow up and improve patient outcomes by identifying those who have missed appointments and encouraging them to return to care.

Each of the interventions, while implemented as a package of services, is delivered to a unique population within each clinic: patients newly eligible for ART (FTIC), patients stable on ART (DMD or AC), patients with poor adherence (EAC), and patients lost from care (TRIC). Patients who are stable on ART can be provided with either decentralized medication delivery or adherence clubs, but not both. These two interventions were offered to stable patients in an effort to provide patients with multiple options for where and how to seek care while reducing the congestion in clinics and increasing the convenience

For peer review only

for patients. Because the patient populations differ, the study can estimate the effect of each intervention individually, in the context of implementation of the overall minimum package.

In order for the study to be as close as possible to routine care conditions the interventions for this study are being implemented by the study sites with no input or oversight from the study team. The interventions follow the National Adherence Guidelines and NDOH has organized trainings prior to the implementation of the interventions to support appropriate implementation of the guidelines.

Selection and Randomization of Study Sites

The evaluation is being conducted at 24 primary health care clinics (PHCs) in South Africa. All study sites follow the current guidelines for HIV care and treatment, dated December 2014[14] . Six clinics were chosen from one district each in Gauteng, KwaZulu-Natal, Limpopo, and North West Provinces. These provinces were chosen in consultation with NDOH to represent high HIV burden regions with high burden districts and high volume clinics. The study team developed a list of all sites in each participating province that met these criteria and selected three matched pairs of clinics per province. Pairs were matched on ART patient volume (1000-1999, 2000-4999, or ≥5000 current ART patients), setting (urban, informal settlement, or rural), location (pairs should be located relatively nearby one another), and HIV viral suppression rate (see Table 2). In each pair, one clinic was randomly assigned (using a computer generated randomization) to receive early implementation of the minimum package of interventions, while the other continued to provide standard of care. No blinding was used.

For peer review only - http://bmjopen.bmj.com/site/about/guidelines.xhtml

Inclusion and Exclusion Criteria

For each objective, we enrolled a specific cohort of patients as shown in Figure 1. All cohorts included patients aged 18 years or above and excluded patients who are not resident in the facility's catchment area, were recorded as having an intention to transfer care to a different facility within 12 months, or were pregnant and eligible for PMTCT. Each cohort had specific inclusion criteria related to eligibility for the specific intervention. These criteria followed the December 2014 national guidelines for HIV care and ART[14] and July 2016 National Adherence Guidelines for Chronic Disease (HIV, TB and NCDs) [11]. In order to identify eligible patients to enroll, we first identified all patients eligible for each intervention based on information recorded on their electronic medical record. At intervention sites, lists were reviewed against clinic records, registers, and other documentation for each intervention, to identify eligible patients. At control sites, we reviewed lists against clinic records to confirm eligibility. If the patient file was found and eligibility for a cohort was confirmed then patients were enrolled sequentially until the required sample size was reached for that cohort. Due to delays in electronic data capturing data was not complete. To account for this at some sites, individuals receiving each intervention were identified directly from registers for that intervention. Clinic files were then reviewed to confirm eligibility and patients were enrolled up until the required sample size was achieved. For each patient enrolled, regardless of the method used to identify them, patient files were reviewed and information was extracted using an electronic case report form to confirm patients did meet all eligibility criteria for that cohort.

Duration of Follow Up

The study enrollment period began on 20 June 2016 and was completed on 16 December 2016. For

For peer review only - http://bmjopen.bmj.com/site/about/guidelines.xhtml

individuals, observation began on the date of determination of eligibility for an intervention. Follow up of the cohorts is now ongoing and is anticipated to be completed in December of 2017. Passive follow up through medical record and database review will continue for a minimum of 14 months after the date of enrollment (two additional months beyond twelve months to allow one-year outcomes to occur and be recorded). This will allow all subjects sufficient follow up time to complete each of the primary outcomes designated.

Data Sources

As noted, this study is relying on routinely collected data for study outcomes. Routine data sources will include TIER.Net, the National Health Laboratory Service (NHLS) database which contains all laboratory tests done in public-sector clinics, and data sets created by entering information from clinic registers, adherence plans, and patient clinic files into a database. Various degrees of strengthening of existing data collection procedures were needed at the facilities in order to ensure complete entries into existing clinic registers or patient files, complete and accurate entry of source data onto electronic files, and the use of a consistent clinic-level patient identifier to link patients between data sources (e.g. a register containing a row for each visit and a patient file containing documents pertaining to that patient will each contribute to the evaluation record for that patient).

Study Outcomes

Table 3 and Figure 1 list the primary and secondary outcomes we will measure for each of the objectives. Each primary outcome includes both a short-term (S) outcome and a final (F) outcome for assessment of the immediate and longer-term effects of the intervention. Short-term outcomes are

For peer review only - http://bmjopen.bmj.com/site/about/guidelines.xhtml

For peer review only

typically focused on retention-based outcomes within the first three to four months after the intervention, with the exception of FTIC in which we assess the impact on treatment initiation within the first month after eligibility. Final outcomes are focused mainly on retention and viral suppression at twelve months. Note that viral suppression in the current South African ART guidelines is defined as viral load below 400 copies/ml[3], and this is the threshold that South Africa's National Health Laboratory Service reports.

For all outcomes, retention in care is defined as (1-% attrition), with attrition calculated as the sum of reported deaths, loss to follow up, and reported transfers to other facilities. Retention will thus be interpreted as "retained in care at facility," since the outcomes of patients who transfer will not be known. Loss to follow up is defined as failure to attend the clinic within 90 days of a scheduled appointment, as stated in the Adherence Guidelines.

Sample Size

Table 4 shows the sample size that is required to detect meaningful differences for Objectives 1-5. Sample sizes were determined using PASS software for cluster-randomized designs. Each sample size was determined to measure our short-term outcome for the objective. All calculations assume a site-clustered design with the clinic as the cluster and 24 clusters evenly split between intervention and comparison groups. We assumed power of 80% and an alpha of 0.05. Sample sizes accounted for the cluster randomized design by assuming a coefficient of variation of 0.1. Each sample size was calculated assuming a baseline proportion of patients achieving the outcome in the absence of the intervention as determined from the literature or experience. Sample sizes were calculated based on being able to detect an absolute increase on outcomes deemed to be clinically meaningful, ranging from 15% to 20%

For peer review only

as determined by consensus of the investigators. The total sample size was calculated to be 3,456 including all of the five HIV cohorts.

Data Analysis

Our general analytic plan will be the same for each of the five interventions. We will begin with descriptive analyses of the characteristics of each of the cohorts stratified by intervention/non-intervention (comparison) status. We will also look for differences within the randomized matched pairs. Because the data will be collected as part of a clustered design, the data analysis will need to account for clustering. For each primary outcome described above, we will conduct a crude analysis comparing the proportion of subjects with the outcome in the intervention and comparison arms. Next, we will conduct an analysis for each outcome accounting only for clustering using generalized estimating equations (GEE) with an unstructured correlation matrix and clustering by treatment site. In all cases, the outcomes are dichotomous and therefore we will calculate relative risks or risk differences comparing the intervention to the comparison arms using a log (or identify) link function and a binomial distribution and will adjust for matched pairs. Next, should any imbalances between treatment groups be detected, we will adjust for those covariates in our GEE model using covariate adjustment. We will look for differences in the effects of the strategies by important baseline characteristics (e.g. size of the treatment population, rural vs. urban, province, etc.) using stratified analyses. In addition, as we are using routine data collection for this study and because much of the outcome data is in electronic data going back to before the period of the intervention, we will also be able to adjust for baseline imbalances using difference-in-differences analyses.

For peer review only - http://bmjopen.bmj.com/site/about/guidelines.xhtml

Ethics and Dissemination

The study has received ethics approval from both the University of the Witwatersrand Human Research Ethics Committee (Medical) and the Boston University Institutional Review Board. In South Africa, we have also received national, provincial, and district-level approvals and the trial has been registered at clinicaltrials.gov (NCT02536768). Results of the study will be presented to key stakeholders as well as at international conferences and published in peer-reviewed journals.

The study team does not have any interaction with study subjects. Data for the study is drawn from existing records that are routinely collected at the study sites as part of routine patient care. We therefore believe that our study poses no physical risks to subjects. The only risk that we believe is posed by this study is that of loss of confidentiality. We are collecting data indicating individuals' HIV status and other sensitive health information. A high level of stigmatization continues to inhibit the disclosure of HIV status in the study population. A breach of confidentiality, for example through inadvertent loss of a storage device or paper files, would thus pose a risk to subjects.

We are protecting against the risk and repercussions of loss of confidentiality in two main ways. First, patient identifiers are stored separately from all other individual data in encrypted, password protected files. Analytic data sets will not contain any identifiers, and the linking files containing the identifiers will be destroyed once all linking has been accomplished. Second, all study data is stored in secure locations. Password-protected laptops and tablets used on site are kept in locked and secure locations when not in use. All data collected on tablets is immediately uploaded to a secure cloud server as soon as data collection for a patient is complete and is not kept on the tablets. Patient data extracted from electronic patient systems is extracted in a password protected double-encrypted format and uploaded to a secure

For peer review only - http://bmjopen.bmj.com/site/about/guidelines.xhtml

server via a dedicated secure virtual private network. All study staff have been trained in Good Clinical Practice, Research Ethics, and study procedures to ensure that they understand both research confidentiality requirements and study confidentiality procedures. Study investigators monitor data collection on an ongoing basis.

We are not seeking informed consent for this study, which is a record review only and poses minimal risk to study subjects. The interventions have been provided by the study clinics as standard care under the early roll-out of NDOH's new Adherence Guidelines, not as part of the study itself.

Dissemination of Findings

The primary audience for this evaluation is the South African National Department of Health and its partners, which will use the results to improve, target, and budget for the national implementation of the Adherence Guidelines. Many of the findings, however, will likely be of broader interest in South Africa and other countries, where effective strategies for improving chronic disease medication adherence are eagerly sought. Results of the evaluation will be made as widely available as possible, through journals, websites, and conferences. Only aggregated, stratified data will be presented and it will not be possible to identify any individual patients from any of the data that is presented.

For peer review only

For peer review only - http://bmjopen.bmj.com/site/about/guidelines.xhtml

REFERENCES

1. SANAC and National Department of Health. South Africa's National Strategic Plan for HIV, TB and STIs 2017-2022. Pretoria, South Africa; 2017.
2. Rosen S, Fox MP. Retention on antiretroviral therapy in South Africa: evidence from a systematic review 2008-2013. Johannesburg; 2014. Report No.: 8.
3. Fox MP, Rosen S. Retention of Adult Patients on Antiretroviral Therapy in Low- -and Middle- -Income Countries: Systematic Review 2008. J Acquir Immune Defic Syndr. 2015;69: 98–108. doi:10.1097/QAI.0000000000000553
4. Fox MP, Shearer K, Maskew M, Meyer-Rath G, Clouse K, Sanne I. Attrition through Multiple Stages of Pre-Treatment and ART HIV Care in South Africa. PLoS One. 2014;9: e110252. doi:10.1371/journal.pone.0110252
5. Clouse K, Pettifor AE, Maskew M, Bassett J, Rie A Van, Behets F, et al. Patient Retention From HIV Diagnosis Through One Year on Antiretroviral Therapy at a Primary Health Care Clinic in Johannesburg , South Africa. J Acquir Immune Defic Syndr. 2013;62: 39–46.
6. DOH. Health Indicators Update- Antiretroviral Indictaors 2013. Pretoria; 2013.
7. National Department of Health. National adherence guidelines for chronic diseases (HIV, TB and NCDs), Version: 7 April 2015. Pretoria; 2015.
8. National Department of Health Republic of South Africa. Standard Operating Procedures for Minimum Package of Interventions to Suport Linkage to Care, Adherence and Retention in Care, Adherence Guidelines for HIV, TB and NCDs [Internet]. Pretoria, South Africa; 2016. Available: http://www.differentiatedcare.org/Portals/0/adam/Content/_YiT3_-qmECUkmkpQvZAIA/File/SOP A5 booklet 20-05-2016.pdf
9. World Bank, The World Bank. Evaluation of interventions to increase the proportion of people living with HIV who are diagnosed, initiated on, adhering to and retained in HIV treatment and care in South Africa. Formative Qualitative Research: Phase 1 Report. 2014.
10. Chaiyachati KH, Ogbuoji O, Price M, Suthar AB, Negussie EK, Bärnighausen T. Interventions to improve adherence to antiretroviral therapy: a rapid systematic review. AIDS. 2014;28 Suppl 2: S187-204. doi:10.1097/QAD.0000000000000252
11. Govindasamy D, Meghij J, Negussi EK, Baggaley RC, Ford N, Kranzer K. Interventions to improve or facilitate linkage to or retention in pre-ART (HIV) care and initiation of ART in low- and middle-income settings: a systematic review. J Int AIDS Soc. 2014;17: 19032.
12. Médecins Sans Frontières Khayelitsha. ART/TB/PMTCT initiation patient education and counselling model report and toolkit. Cape Town; 2015.
13. Medicins Sans Frontieres. ART adherence club report and toolkit. Cape Town; 2014.
14. National Department of Health. National consolidated guidelines for the prevention of mother-to-child transmission of HIV (PMTCT) and the management of HIV in children, adolescents and adults. Pretoria; 2014.
15. Rosen S, Maskew M, Fox MP, Nyoni C, Mongwenyana C, Malete G, et al. Initiating Antiretroviral Therapy for HIV at a Patient's First Clinic Visit: The RapIT Randomized Controlled Trial. Binagwaho A, editor. PLOS Med. 2016;13: e1002015. doi:10.1371/journal.pmed.1002015
16. Fox M, Maskew M, MacPhail A. Cohort Profile: The Themba Lethu Clinical Cohort, Johannesburg, South Africa. Int J Epidemiol. 2013;42: 430–439. doi:10.1093/ije/dys029
17. Fox M, Shearer K, Maskew M, Macleod W, Majuba P, Macphail P, et al. Treatment outcomes after 7 years of public-sector HIV treatment. AIDS. 2012;26: 1823–8. doi:10.1097/QAD.0b013e328357058a
18. Fox MP, Maskew M, Brennan AT, Evans D, Onoya D, Malete G, et al. Cohort profile: the Right to Care Clinical HIV Cohort, South Africa. BMJ Open. 2017;7: bmjopen-2016-015620. doi:10.1136/bmjopen-2016-015620

For peer review only - http://bmjopen.bmj.com/site/about/guidelines.xhtml

Authors' contributions: NF, MG, MP, SP, SR and MPF all contributed to developing the protocol. AH, JM, DW, MN and YP all contributed substantive changes to the protocol. MPF drafted the manuscript. All authors were involved in editing the final manuscript.

Funding statement: This work was supported by World Bank trust funds from several governments and Government of South Africa domestic health financing.

Competing interests statement: The authors declare that they have no competing interests, as the study funders played no role in the decision to publish this protocol.

For peer review only

Table 1. South Africa's National Adherence Guidelines minimum package of interventions

Approach	Intervention
Education and counselling	1. Fast track initiation counseling*<br>2. Enhanced adherence counseling for unstable patients*<br>3. Child disclosure counseling for children living with HIV
Repeat prescription collection strategies	4. Adherence clubs*<br>5. Spaced and fast lane appointment systems<br>6. Decentralised medication delivery*
Patient tracing	7. Early tracing of all missed appointments*
Integrated HIV, TB, NCD care	8. Integrated consultation and counselling

*Indicates interventions included in this evaluation

For peer review only

Table 2: Location of early learning sites, allocation status and value of matching variables used to determine matched pairs*

District & Province	Pair	Site number	Sub-district [1]	Study allocation	Total remaining on ART (Nov 2014)	% of viral loads where VL<400 copies/ml [2]
Ekurhuleni, Gauteng	1	1	S2	Intervention	1094	71%
		2	S2	Control	1098	66%
	2	3	S2	Intervention	2676	86%
		4	S2	Control	2749	76%
	3	5	S2	Intervention	1929	84%
		6	S2	Control	1072	80%
Mopani, Limpopo	4	7	Greater Tzaneen	Intervention	1720	71%
		8	Greater Tzaneen	Control	1022	64%
	5	9	Greater Giyani	Intervention	1702	73%
		10	Greater Giyani	Control	1445	76%
	6	11	Greater Tzaneen	Intervention	1370	77%
		12	Greater Tzaneen	Control	1027	76%
Bojanala Platinum, North West	7	13	Madibeng	Intervention	4147	83%
		14	Madibeng	Control	4182	82%
	8	15	Madibeng	Intervention	1152	83%
		16	Madibeng	Control	1224	81%
	9	1	Rustenburg	Intervention	3951	80%

For peer review only - http://bmjopen.bmj.com/site/about/guidelines.xhtml

		7				
		18	Rustenburg	Control	3328	78%
King Cetshwayo (previously uThungulu), KwaZulu Natal	10	19	uMlalazi	Intervention	1900	72%
		20	uMlalazi	Control	1053	69%
	11	21	uMhlathuze	Intervention	5037	83%
		22	uMhlathuze	Control	7305	82%
	12	23	Ntambanana	Intervention	1111	81%
		24	Ntambanana	Control	1184	88%

[1] Used as proxy for setting and location

[2] NHLS data April 2014 to March 2015

* Data source: MacLeod, W., Bor, J., Crawford, K., & Carmona, S. (2015). Analysis of Big Data for better targeting of ART Adherence Strategies: Spatial clustering analysis of viral load suppression by South African province, district, sub-district and facility (April 2014-March 2015). Department of Health, Pretoria, South Africa.

For peer review only - http://bmjopen.bmj.com/site/about/guidelines.xhtml

Table 3. Short-term (S) and final (F) evaluation outcomes for the Adherence Guideline impact evaluation in South Africa

Objective	Primary Outcome	Secondary outcomes
Fast track ART initiation counseling (Objective/Cohort 1)	Proportion of patients who initiate ART within 30 days of becoming ART eligible (S) and the proportion of patients who are alive, in care, and virally suppressed (< 400 copies/ml^3) within nine months of ART eligibility (F).	Proportion of patients who initiate ART within one week of becoming ART eligible<br><br>Demographic and clinical characteristics of patients who do and do not achieve primary outcomes (age, sex, baseline CD4 counts, TB diagnosis, other characteristics as allowed by data).
Adherence clubs (Objective/Cohort 2)	Proportion of patients eligible for participation in an adherence club who receive all medications within the first three months after club eligibility (S) and the proportion virally suppressed (< 400 copies/ml^3) at twelve months after club eligibility (F).	Proportion of patients consistently participating in club<br><br>Demographic and clinical characteristics of patients who do and do not achieve primary outcomes.
Decentralized medication delivery (Objective/Cohort 3)	Proportion of patients eligible for decentralized medication delivery who receive all medications within the first three (S) months after delivery eligibility and viral suppression (< 400 copies/ml^3) twelve months after delivery eligibility (F).	Proportion of patients consistently receiving medications<br><br>Demographic and clinical characteristics of patients who do and do not achieve primary outcomes.
Enhanced adherence counseling (Objective/Cohort 4)	Proportion of patients with an elevated viral load who are alive, retained in care and resuppress their viral load (< 400copies/ml^3) within three (S) and twelve months (F) of eligibility for enhanced adherence counseling.	Demographic and clinical characteristics of patients who do and do not achieve primary outcomes.
Early tracing of patients lost to follow up (Objective/Cohort 5)	Proportion of patients eligible for early patient tracing who return to care within three (S) and twelve (F) months of eligibility.	Proportion of patients reached by tracers<br><br>Number of tracing attempts required; proportion of patients retained in care for at least one additional routine visit after tracing<br><br>Demographic and clinical characteristics of patients who do and do not achieve primary outcomes.

For peer review only - http://bmjopen.bmj.com/site/about/guidelines.xhtml

Table 4. Sample sizes for each objective of the Adherence Guideline impact evaluation study in South Africa

Objective	Sample Size	Rationale
Objective 1—Fast Track ART Initiation Counseling	720 patients	The RapIT study of rapid ART initiation[15], conducted at a well-managed PHC in Gauteng Province, found that about 60% of ART-eligible patients initiated under standard care within 30 days. Conservatively assuming 60% initiation without the intervention and 75% with the intervention, 30 subjects in each of the 24 clusters for 720 total subjects will be required to detect a difference of 15%. We have increased this by 20% to account for ineligible patients.
Objective 2—Adherence Clubs	576 patients	Data from Themba Lethu Clinic[16–18] show that about 80% of patients made all of their medication pickups over a three month period. It is anticipated that 24 subjects per clinic for a total of 576 patients will be needed to detect a difference of 15%. We have increased this by 20% to account for ineligible patients.
Objective 3—Decentralized Medication Delivery	576 patients	Data from Themba Lethu Clinic[16–18] show about 80% of patients made all of their medication pickups over a three month period. It is anticipated that 24 subjects per clinic for a total of 576 patients will be needed to detect a difference of 15%. We have increased this by 20% to account for ineligible patients.
Objective 4—Enhanced Adherence Counseling	1008 patients	Data from KwaZulu-Natal Province indicate that 52% of patients with a detectable viral load re-suppress after one session. It is anticipated that 42 subjects per clinic for a total of 1008 patients will be needed to detect a difference of 15%. We have increased this by 20% to account for ineligible patients.
Objective 5—Early tracing of patients lost to follow up	576 patients	Data from various Right to Care clinics[18] suggest that the proportion of patients who are lost from care who return with no or little intervention is low, between 20-35%. It is anticipated that 24 subjects per clinic for a total of 576 patients will be needed to detect a difference of 15% assuming a baseline of 30% loss to follow up without intervention. We have increased this by 20% to account for ineligible patients.

For peer review only

For peer review only - http://bmjopen.bmj.com/site/about/guidelines.xhtml

Figure Legends

Figure 1 – Eligible population for each evaluation cohort and short-term and final endpoints for the impact evaluation

For peer review only

For peer review only - http://bmjopen.bmj.com/site/about/guidelines.xhtml

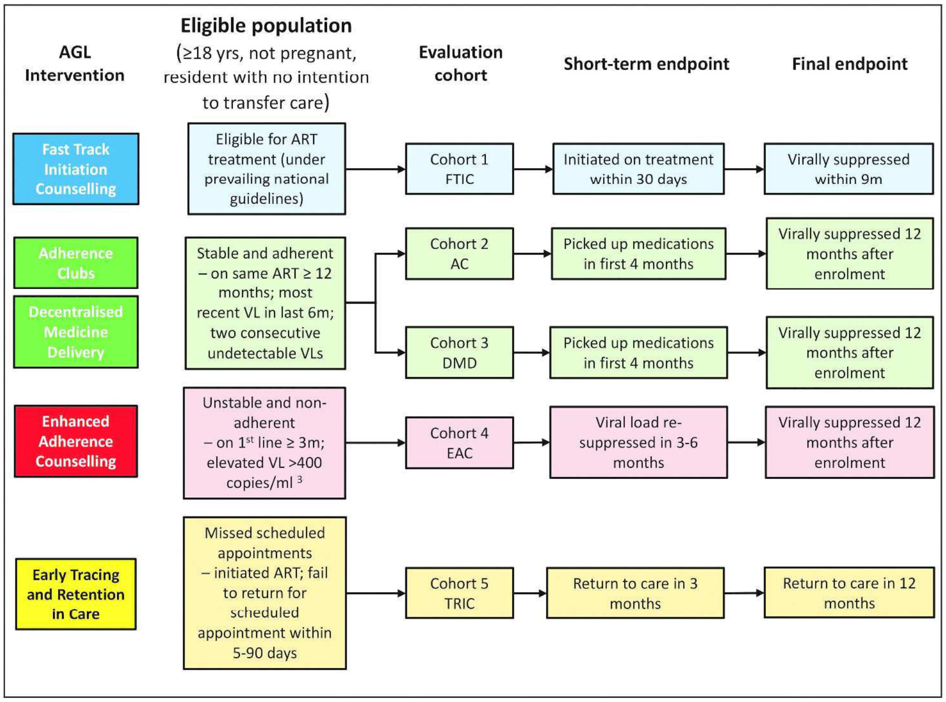

Figure 1 – Eligible population for each evaluation cohort and short-term and final endpoints for the impact evaluation

254x190mm (300 x 300 DPI)

w only

STANDARD PROTOCOL ITEMS: RECOMMENDATIONS FOR INTERVENTIONAL TRIALS

SPIRIT 2013 Checklist: Recommended items to address in a clinical trial protocol and related documents*

Section/item	Item No	Description	Addressed on page number
**Administrative information**			
Title	1	Descriptive title identifying the study design, population, interventions, and, if applicable, trial acronym	______1_______
Trial registration	2a	Trial identifier and registry name. If not yet registered, name of intended registry	______13______
	2b	All items from the World Health Organization Trial Registration Data Set	______Multiple__
Protocol version	3	Date and version identifier	_Protocol available
Funding	4	Sources and types of financial, material, and other support	Funding statement 18
Roles and responsibilities	5a	Names, affiliations, and roles of protocol contributors	1
	5b	Name and contact information for the trial sponsor	1
	5c	Role of study sponsor and funders, if any, in study design; collection, management, analysis, and interpretation of data; writing of the report; and the decision to submit the report for publication, including whether they will have ultimate authority over any of these activities	_18__________
	5d	Composition, roles, and responsibilities of the coordinating centre, steering committee, endpoint adjudication committee, data management team, and other individuals or groups overseeing the trial, if applicable (see Item 21a for data monitoring committee)	__n/a_________

For peer review only

For peer review only - http://bmjopen.bmj.com/site/about/guidelines.xhtml

Introduction

Background and rationale	6a	Description of research question and justification for undertaking the trial, including summary of relevant studies (published and unpublished) examining benefits and harms for each intervention	____4-5_____
	6b	Explanation for choice of comparators	____6-8_____
Objectives	7	Specific objectives or hypotheses	____4-5______
Trial design	8	Description of trial design including type of trial (eg, parallel group, crossover, factorial, single group), allocation ratio, and framework (eg, superiority, equivalence, noninferiority, exploratory)	_____6_______

Methods: Participants, interventions, and outcomes

Study setting	9	Description of study settings (eg, community clinic, academic hospital) and list of countries where data will be collected. Reference to where list of study sites can be obtained	____4 and 6__
Eligibility criteria	10	Inclusion and exclusion criteria for participants. If applicable, eligibility criteria for study centres and individuals who will perform the interventions (eg, surgeons, psychotherapists)	______9-10____
Interventions	11a	Interventions for each group with sufficient detail to allow replication, including how and when they will be administered	________6-8___
	11b	Criteria for discontinuing or modifying allocated interventions for a given trial participant (eg, drug dose change in response to harms, participant request, or improving/worsening disease)	____n/a______
	11c	Strategies to improve adherence to intervention protocols, and any procedures for monitoring adherence (eg, drug tablet return, laboratory tests)	____ n/a ______
	11d	Relevant concomitant care and interventions that are permitted or prohibited during the trial	_____ n/a ____
Outcomes	12	Primary, secondary, and other outcomes, including the specific measurement variable (eg, systolic blood pressure), analysis metric (eg, change from baseline, final value, time to event), method of aggregation (eg, median, proportion), and time point for each outcome. Explanation of the clinical relevance of chosen efficacy and harm outcomes is strongly recommended	_____11-12____
Participant timeline	13	Time schedule of enrolment, interventions (including any run-ins and washouts), assessments, and visits for participants. A schematic diagram is highly recommended (see Figure)	____9-10______

For peer review only - http://bmjopen.bmj.com/site/about/guidelines.xhtml

Sample size	14	Estimated number of participants needed to achieve study objectives and how it was determined, including clinical and statistical assumptions supporting any sample size calculations	___12-13_____
Recruitment	15	Strategies for achieving adequate participant enrolment to reach target sample size	____13______
**Methods: Assignment of interventions (for controlled trials)**			
Allocation:			
Sequence generation	16a	Method of generating the allocation sequence (eg, computer-generated random numbers), and list of any factors for stratification. To reduce predictability of a random sequence, details of any planned restriction (eg, blocking) should be provided in a separate document that is unavailable to those who enrol participants or assign interventions	_____9______
Allocation concealment mechanism	16b	Mechanism of implementing the allocation sequence (eg, central telephone; sequentially numbered, opaque, sealed envelopes), describing any steps to conceal the sequence until interventions are assigned	_____n/a____
Implementation	16c	Who will generate the allocation sequence, who will enrol participants, and who will assign participants to interventions	_____9______
Blinding (masking)	17a	Who will be blinded after assignment to interventions (eg, trial participants, care providers, outcome assessors, data analysts), and how	_____9______
	17b	If blinded, circumstances under which unblinding is permissible, and procedure for revealing a participant's allocated intervention during the trial	______n/a____
**Methods: Data collection, management, and analysis**			
Data collection methods	18a	Plans for assessment and collection of outcome, baseline, and other trial data, including any related processes to promote data quality (eg, duplicate measurements, training of assessors) and a description of study instruments (eg, questionnaires, laboratory tests) along with their reliability and validity, if known. Reference to where data collection forms can be found, if not in the protocol	_____13______
	18b	Plans to promote participant retention and complete follow-up, including list of any outcome data to be collected for participants who discontinue or deviate from intervention protocols	_____n/a_____

For peer review only - http://bmjopen.bmj.com/site/about/guidelines.xhtml

Data management	19	Plans for data entry, coding, security, and storage, including any related processes to promote data quality (eg, double data entry; range checks for data values). Reference to where details of data management procedures can be found, if not in the protocol	_____11,13____
Statistical methods	20a	Statistical methods for analysing primary and secondary outcomes. Reference to where other details of the statistical analysis plan can be found, if not in the protocol	____13_____
	20b	Methods for any additional analyses (eg, subgroup and adjusted analyses)	____13______
	20c	Definition of analysis population relating to protocol non-adherence (eg, as randomised analysis), and any statistical methods to handle missing data (eg, multiple imputation)	_____n/a_____
**Methods: Monitoring**			
Data monitoring	21a	Composition of data monitoring committee (DMC); summary of its role and reporting structure; statement of whether it is independent from the sponsor and competing interests; and reference to where further details about its charter can be found, if not in the protocol. Alternatively, an explanation of why a DMC is not needed	_____ n/a _____
	21b	Description of any interim analyses and stopping guidelines, including who will have access to these interim results and make the final decision to terminate the trial	_____ n/a _____
Harms	22	Plans for collecting, assessing, reporting, and managing solicited and spontaneously reported adverse events and other unintended effects of trial interventions or trial conduct	______ n/a ____
Auditing	23	Frequency and procedures for auditing trial conduct, if any, and whether the process will be independent from investigators and the sponsor	______ n/a ____
**Ethics and dissemination**			
Research ethics approval	24	Plans for seeking research ethics committee/institutional review board (REC/IRB) approval	_____13_____
Protocol amendments	25	Plans for communicating important protocol modifications (eg, changes to eligibility criteria, outcomes, analyses) to relevant parties (eg, investigators, REC/IRBs, trial participants, trial registries, journals, regulators)	_____13-14__

For peer review only - http://bmjopen.bmj.com/site/about/guidelines.xhtml

Consent or assent	26a	Who will obtain informed consent or assent from potential trial participants or authorised surrogates, and how (see Item 32)	___n/a______
	26b	Additional consent provisions for collection and use of participant data and biological specimens in ancillary studies, if applicable	_____ n/a_____
Confidentiality	27	How personal information about potential and enrolled participants will be collected, shared, and maintained in order to protect confidentiality before, during, and after the trial	_____14______
Declaration of interests	28	Financial and other competing interests for principal investigators for the overall trial and each study site	_____18______
Access to data	29	Statement of who will have access to the final trial dataset, and disclosure of contractual agreements that limit such access for investigators	___data agreement___
Ancillary and post-trial care	30	Provisions, if any, for ancillary and post-trial care, and for compensation to those who suffer harm from trial participation	____n/a_______
Dissemination policy	31a	Plans for investigators and sponsor to communicate trial results to participants, healthcare professionals, the public, and other relevant groups (eg, via publication, reporting in results databases, or other data sharing arrangements), including any publication restrictions	____15_______
	31b	Authorship eligibility guidelines and any intended use of professional writers	____n/a_______
	31c	Plans, if any, for granting public access to the full protocol, participant-level dataset, and statistical code	Declarations
**Appendices**			
Informed consent materials	32	Model consent form and other related documentation given to participants and authorised surrogates	n/a
Biological specimens	33	Plans for collection, laboratory evaluation, and storage of biological specimens for genetic or molecular analysis in the current trial and for future use in ancillary studies, if applicable	n/a

*It is strongly recommended that this checklist be read in conjunction with the SPIRIT 2013 Explanation & Elaboration for important clarification on the items. Amendments to the protocol should be tracked and dated. The SPIRIT checklist is copyrighted by the SPIRIT Group under the Creative Commons "Attribution-NonCommercial-NoDerivs 3.0 Unported" license.

For peer review only - http://bmjopen.bmj.com/site/about/guidelines.xhtml
